# Kinetic and structural changes in *H* *smt* PheRS, induced by pathogenic mutations in human *FARS* *2*

**DOI:** 10.1002/pro.3176

**Published:** 2017-05-03

**Authors:** Ekaterine Kartvelishvili, Dmitry Tworowski, Hilary Vernon, Nina Moor, Jing Wang, Lee‐Jun Wong, Zofia Chrzanowska‐Lightowlers, Mark Safro

**Affiliations:** ^1^ Department of Structural Biology Weizmann Institute of Science Israel; ^2^ McKusick‐Nathans Institute of Genetic Medicine Johns Hopkins School of Medicine Baltimore; ^3^ Laboratory of Bioorganic Chemistry of Enzymes Institute of Chemical Biology and Fundamental Medicine Novosibirsk Russia; ^4^ Ambry Genetics California; ^5^ Molecular and Human Genetics, Baylor College of Medicine Houston Texas; ^6^ Wellcome Centre for Mitochondrial Research, Newcastle University Newcastle NE2 4HH United Kingdom

**Keywords:** mitochondrial diseases, mitochondrial PheRS, mutants, X‐ray structures, molecular dynamic simulations, kinetic experiments

## Abstract

Mutations in the mitochondrial aminoacyl‐tRNA synthetases (*mt*aaRSs) can cause profound clinical presentations, and have manifested as diseases with very selective tissue specificity. To date most of the *mt*aaRS mutations could be phenotypically recognized, such that clinicians could identify the affected *mt*aaRS from the symptoms alone. Among the recently reported pathogenic variants are point mutations in *FARS2* gene, encoding the human mitochondrial PheRS. Patient symptoms range from spastic paraplegia to fatal infantile Alpers encephalopathy. How clinical manifestations of these mutations relate to the changes in three‐dimensional structures and kinetic characteristics remains unclear, although impaired aminoacylation has been proposed as possible etiology of diseases. Here, we report four crystal structures of *Hsmt*PheRS mutants, and extensive MD simulations for wild‐type and nine mutants to reveal the structural changes on dynamic trajectories of *Hsmt*PheRS. Using steady‐state kinetic measurements of phenylalanine activation and tRNA^Phe^ aminoacylation, we gained insight into the structural and kinetic effects of mitochondrial disease‐related mutations in *FARS2* gene.

**Abbreviations**
aa: amino acids
ABD: anticodon‐binding domain
CAM: catalytic module
3DN: aa numbering as in 3D structure
*Hsmt*PheRS: human mitochondrial phenylalanyl‐tRNA synthetase
MD: molecular dynamic
WT: wild type.

## Introduction

Mitochondria are vital organelles that are present in all nucleated mammalian cells and possess their own genome and biosynthetic machinery to produce both RNAs and proteins. Of the ∼1500 proteins that comprise a functional mitochondrion, ∼99% of these are nuclear encoded, synthesized on cytosolic ribosomes and imported into the mitochondrial matrix. Amongst these are the mitochondrial *mt*aaRSs, which are key components in protein biosynthesis. Abnormalities in *mt*aaRSs are an increasingly recognized cause of human mitochondrial disease with profound clinical presentations.^1^ Loss of quality control in mitochondrial protein translation is expected to reduce oxidative phosphorylation capacity leading to ATP deficiency in all tissues. In the increasing number of patients reported thus far, the pattern of which tissue is affected is almost always associated with a particular *mt*aaRS, such that clinicians can often identify the affected *mt*aaRS from the symptoms alone.^1^, ^2^ Pathogenic variants have been described in genes encoding 12 of the 19 *mt*aaRSs and are associated with a variety of clinical presentations including leukoencephalopathy with brain stem and spinal cord involvement and lactate elevation (LBSL, MIM 611105) caused by variants in *DARS2*, pontocerebellar atrophy type 6 (MIM 611523) caused by variants in *RARS2*, and a fatal infantile cardiomyopathy (MIM 614096) caused by variants in *AARS2*, among others.^1^, ^2^

The overall prevalence of defects in mitochondrial aaRS2 gene is ∼1% (7/800) among observed 800 patients, who are suspected of having mitochondrial disorders.^3^ It is important to note that among them 57% (four of seven) of positive findings are in the *FARS2* gene.^3^ Pathogenic variants in *FARS2* encoding the human mitochondrial PheRS (*Hsmt*PheRS) have been associated with phenotypes ranging from spastic paraplegia to fatal infantile Alpers encephalopathy.^4^, ^5^, ^6^, ^7^ How clinical manifestation and disease phenotypes of individual mutations in *Hsmt*PheRS, relate to the changes in their 3D‐structures and enzymatic activities has remained unclear, although impaired aminoacylation activity of the aaRSs has been generally proposed as a potential etiology of these diseases.

Correct charging of tRNAs with their cognate amino acid (aa), is a crucial quality control step in protein synthesis. The covalent linking of the correct aa to the relevant tRNA is accomplished by the aaRSs through a two‐step aminoacylation reaction.^8^ Before a chemical reaction will start, the aaRS recognizes and binds both the cognate aa and ATP in their specific binding pockets. In the first step of the reaction, the amino acid is activated by ATP, forming the first intermediate Phe‐AMP. In the second step, the aa moiety is transferred to the 3'‐terminal ribose of the cognate tRNA, generating the final aminoacyl‐tRNA, and releasing AMP.^8^

**Figure 1:**
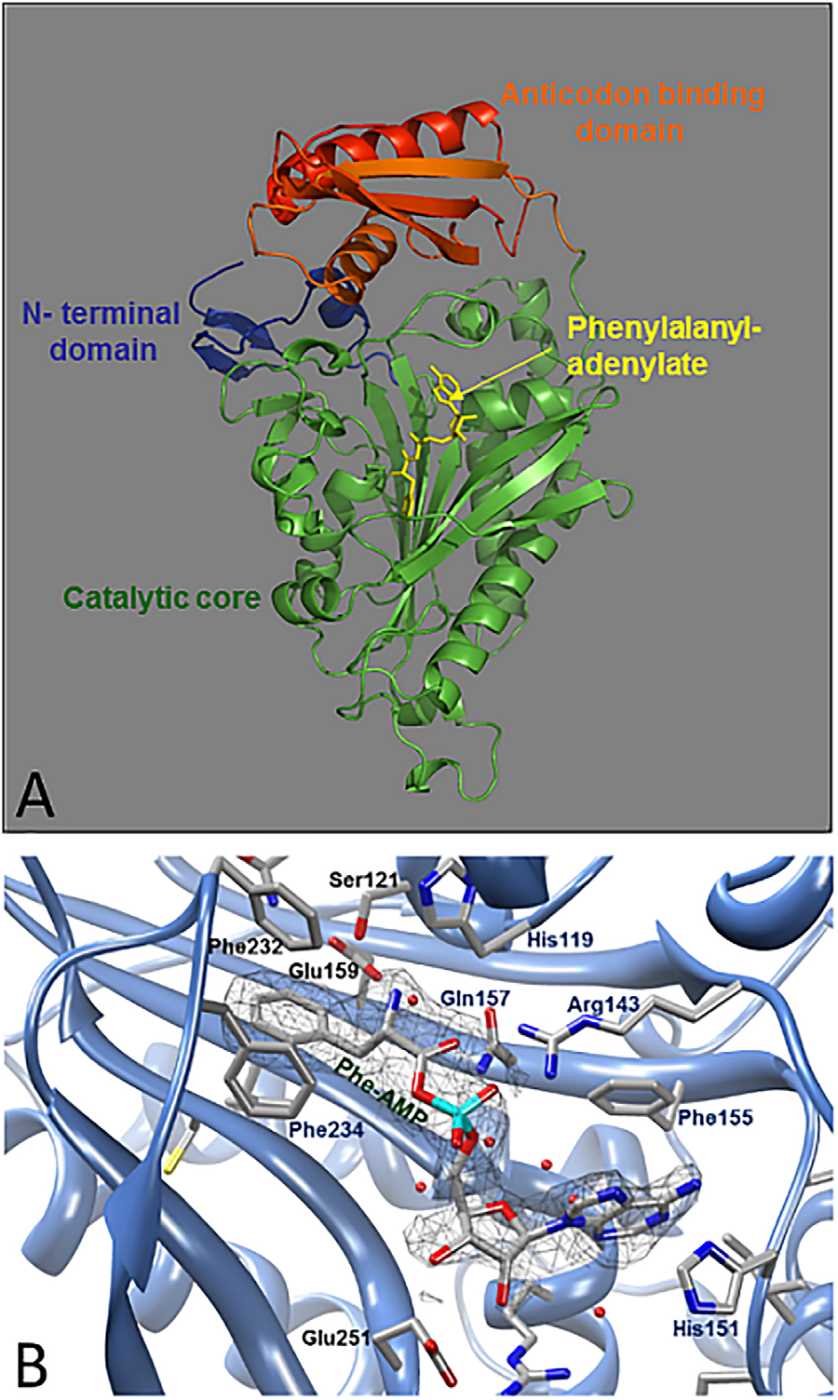
(A) The overall structure of the *Hsmt*PheRS enzyme complexed with PheOH‐AMP is presented. The N‐terminal region (residues 1–47) depicted in dark blue, the catalytic domain (residues 48–289) in green, and the C‐terminal domain (residues 323–415) in red. The ligand (PheOH‐AMP) is shown in ribbon representation (yellow). (B) The unbiased (*F*_obs_ – *F*_calc_) electron density map of phenylalanyl‐adenylate analog (Phe‐AMP) calculated with the phases derived from the nearly complete model of *Hsmt*PheRS and contoured at 2.0*σ*. The protein residues participating in direct and water‐mediated contacts with PheOH‐AMP are shown. Water molecules are marked by red spheres. The anchoring of the α‐ ${\text{NH}}_{3}^{+}$ group of PheOH‐AMP is achieved by direct hydrogen bonding between the O^γ^ atom of Serα121, and the well‐ordered water molecule X9 observed in almost all PheRS complexes. The X9 molecule, in turn, is located at an H‐bonding distance from the O^γ^ of Thr120, the N^ε^ 2 of Gln157, and the O^ε1^ of Glu159.

Eukaryotic cells harbor two different types of phenylalanyl‐tRNA synthetase (PheRS): the heterotetrameric cytosolic and monomeric mitochondrial forms.^9^, ^10^, ^11^, ^12^ The mature *Hsmt*PheRS homolog is a single‐chain enzyme comprising 415 amino acids and crystal structures of this enzyme have now been determined for both the native form and in complexes with various ligands.^13^, ^14^, ^15^ The *Hsmt*PheRS is composed of three major structural blocks: the N‐terminal region, the CAM, and the ABD^10^ [Fig. 1(A)]. Conservation of the topology of the binding site cavity is critical for correct aminoacylation. The bottom surface of the pocket is covered by invariant glycines, thus providing the space required for the phenylalanine (Phe) and ATP moieties^10^, ^16^ [Fig. 1(B)]. One wall is made up entirely of residues that participate in the formation of hydrogen bonds (HB) with the Phe substrate (via amine and carboxyl functional groups) and transition state intermediates: Glu159, Ser121, His119, and Gln157.

Specific recognition of the Phe is achieved by interactions where the substrate phenyl ring and two neighboring phenyl rings of Phe232 and Phe234 make “edge‐to‐face” contacts^17^ [Fig. 1(B)]. The appearance of the Phe substrate in such an environment doubles the attractive potential energy of interaction of a single “edge‐to‐face” aromatic‐aromatic interaction and thus makes the Phe‐PheRS recognition highly specific and very favorable energetically. The anchoring of the Phe substrate amino group is achieved by its interactions with Ser121, His119, and via the well‐ordered water molecule with Gln157 and Glu159.

**Figure 2:**
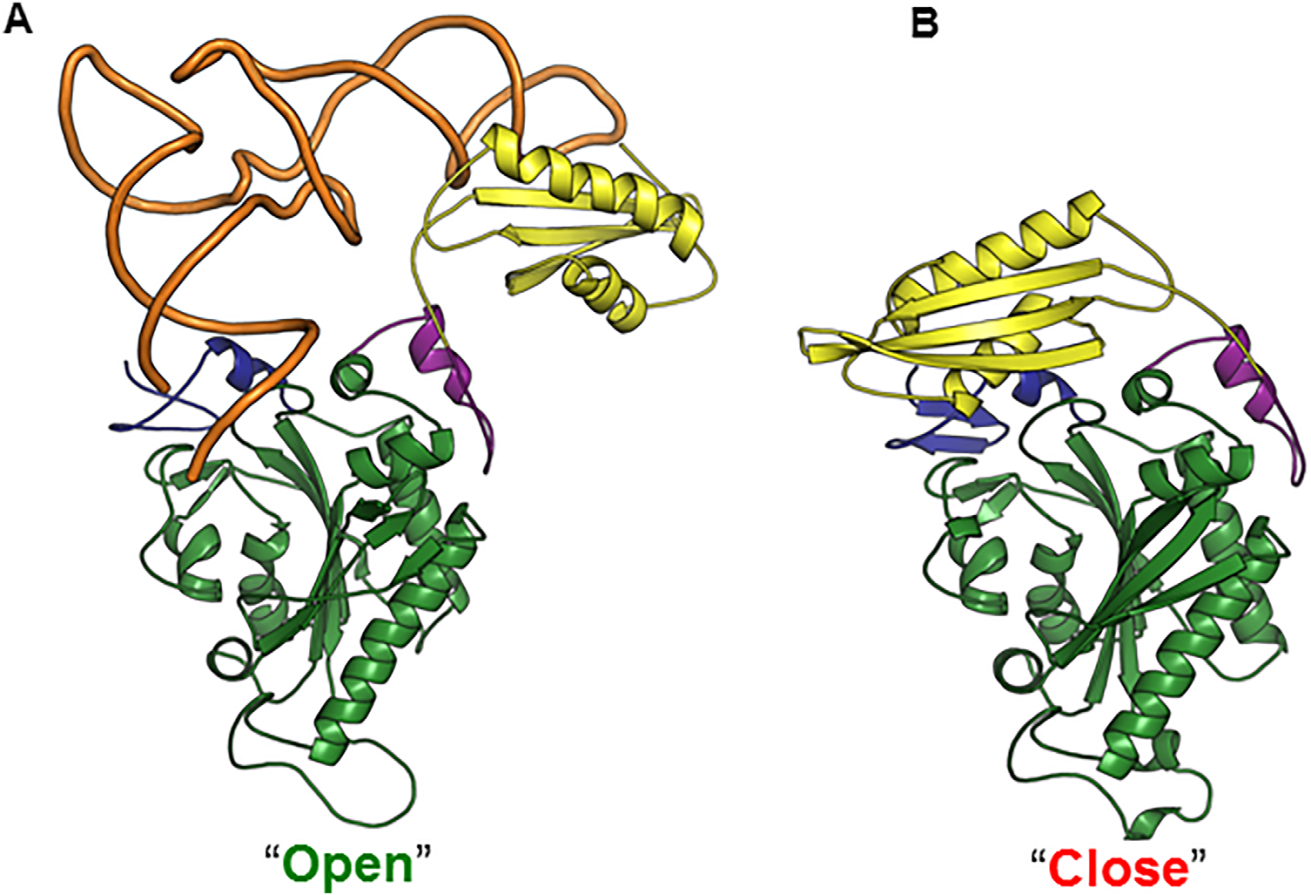
Transitions from the active “open” (A) to inactive “closed” (B) conformations of *Hsmt*PheRS upon complex formation with tRNA^Phe^ are shown with the tRNA^Phe^ molecule depicted by “worm” representation.^12^, ^18^ PDB ID of the *Hsmt*PheRS complexed with tRNA^Phe^ is 3TUP.

The crystal structure of *Hsmt*PheRS‐tRNA^Phe^ complex, biochemical and small‐angle X‐ray scattering data indicate that the formation of the catalytically active complex with tRNA^Phe^ in human mitochondria requires a significant rearrangement of the ABD.^13^, ^18^, ^19^ To bind tRNA^Phe^ correctly, the ABD must undergo a ∼160° hinge‐type rotation upon binding (Fig. 2). Such global repositioning of the domains is tRNA modulated and governed by long range electrostatic interactions.^20^, ^21^ Two basic conformations for *Hsmt*PheRS, have revealed themselves: a “closed” inactive unbound state and an “open” active state upon tRNA binding.^19^ A transition, however, between two basic conformations, happens through the various intermediate (probably short‐live) conformations.

To explore the effects of mutations on the functional activity of *Hsmt*PheRS *in vitro*, we performed steady‐state kinetic measurements for both the Phe activation reactions and tRNA^Phe^ aminoacylation of WT and mutant enzymes. To gain an insight into the problem we solved the crystal structures of four *Hsmt*PheRS mutants. We also ran extensive MD simulations to reveal the structural changes on the dynamic trajectories of *Hsmt*PheRS, caused by reported point mutations.

## Results

### Kinetic study of functional activities of *Hsmt*PheRS mutants

Three of the nine *Hsmt*PheRS variants (Supporting Information Table S1) that we analysed demonstrated modest changes in the catalytic efficiency of charging tRNA^Phe^ (summarized in Table 1). The Pro49Ala and Arg387Gln mutations led to a 1.2‐ to 1.3‐fold decrease in the *k*_cat_/*K*_m_ value due to a reduced *k*_cat_. The Thr210Met replacement increased the catalytic efficiency of tRNA^Phe^ charging by 1.4‐fold due to a lower *K*_m_ value for tRNA^Phe^. Mutations of residues within different structural domains, namely Asp289Tyr and Arg383Cys, halved the catalytic efficiency. In the case of Asp289Tyr, the reduced *k*_cat_/*K*_m_ resulted from a decreased *k*_cat_; whilst for Arg383Cys, it was due to a combination of increased *K*_m_ and decreased *k*_cat_ (Table 1). Combination of increased *K*_m_ value for Phe and a decreased *k*_cat_ caused even more significant effects in the His99Asp and Arg117Gly mutants where a 40‐ to 50‐fold reduction in the *k*_cat_/*K*_m_ value was recorded. The effect was even stronger in two other *Hsmt*PheRS variants, namely His123Pro and Gly273Ser, which demonstrated negligible ability to charge tRNA^Phe^. Only by performing the kinetic measurements at very high concentrations of the mutant enzymes, could we estimate their catalytic efficiencies, which were at least 3100‐fold and 4000‐fold lower than that of the wild‐type enzyme.

**Table 1:** Kinetic Parameters of tRNA^Phe^ Aminoacylation by HsmtPheRS Mutants

*Hsmt*PheRS	Substrate	*K*_m_ (µ*M*)	*k*_cat_ (min^−1^)	*k*_cat_/*K*_m_ (min^−1^ µ*M* ^−1^)	*k*_cat_/*K*_m_ (relative)
Wild‐type	tRNA^Phe^	1.2 ± 0.2	11.5 ± 1.2	9.6	1
Wild‐type	Phe	2.4 ± 0.4	11.5 ± 1.5	4.8	1
P49A	tRNA^Phe^	1.1 ± 0.2	8.3 ± 0.8	7.5	0.78
H99D	Phe	5.6 ± 0.8	0.72 ± 0.14	0.13	0.027
R117G	Phe	12 ± 3	1.2 ± 0.13	0.10	0.021
H123P	tRNA^Phe^				∼0.00032^a^
T210M	tRNA^Phe^	0.36 ± 0.04	4.8 ± 0.4	13.3	1.4
G273S	tRNA^Phe^				∼0.00025^a^
D289Y	tRNA^Phe^	1.0 ± 0.18	5.0 ± 0.5	5.0	0.52
R383C	tRNA^Phe^	2.1 ± 0.3	8.7 ± 0.7	4.1	0.43
R387Q	tRNA^Phe^	1.2 ± 0.1	11 ± 1.2	8.3	0.86

a *k*_cat_/*K*_m_ value was estimated from the initial rate of aminoacylation reaction normalized to the enzyme concentration (1.5 μ*M* for mutant; 0.15 μ*M* for the wild‐type).

**Figure 3:**
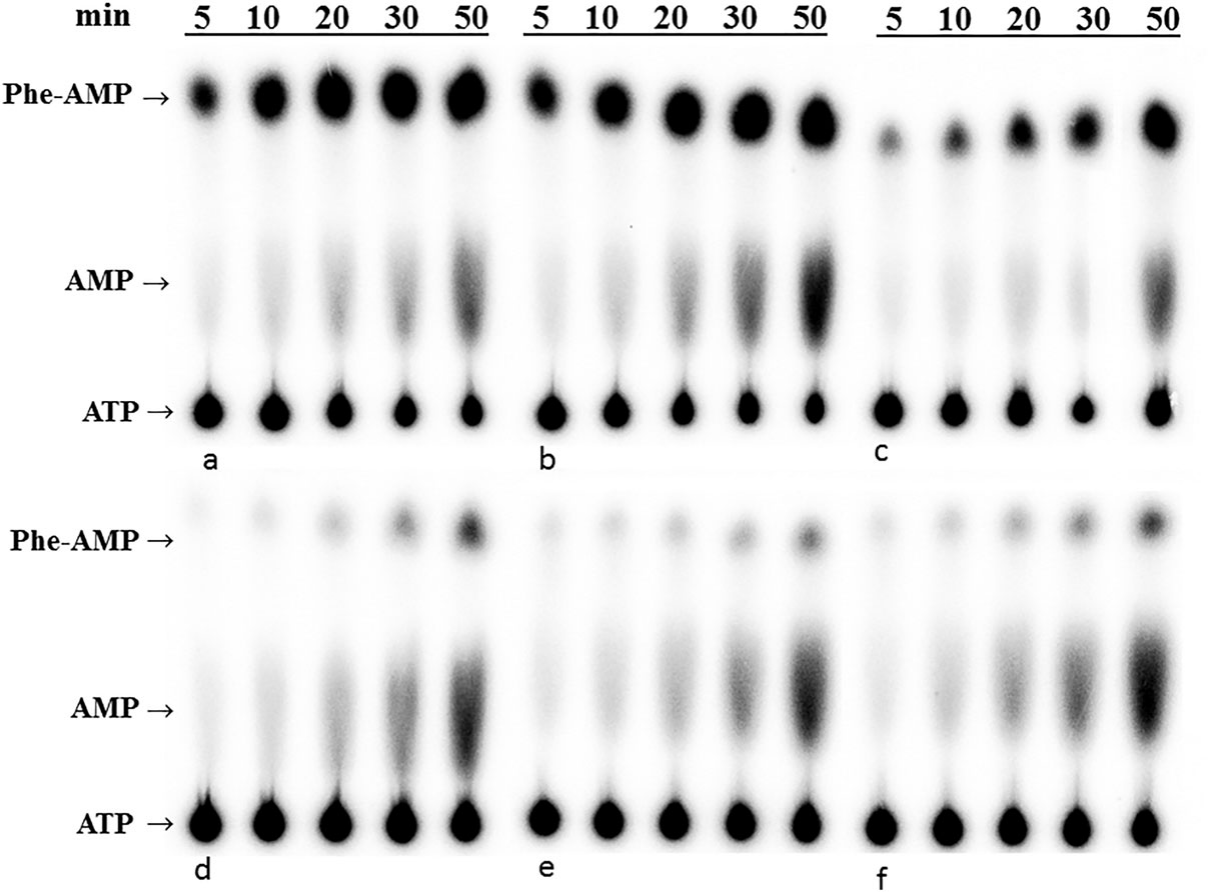
Phenylalanine activation by wild‐type and mutant forms of *Hsmt*PheRS. The time courses of formation of Phe‐adenylate and AMP (produced due to hydrolysis of the adenylate) was analyzed by TLC. The reaction was performed with 20 μ*M* [α‐^32^P]ATP, 9 m*M* MgCl_2_, 150 n*M* WT or mutant PheRSs, 1 m*M* phenylalanine, and 20 U/mL of inorganic pyrophosphatase. These are shown in (A) wild‐type *Hsmt*PheRS; (B) mutant Pro49Ala; (C) mutant His99Asp; (D) mutant Arg117Gly; (E) mutant His123Pro; and (F) mutant Gly273Ser.

Several mutants were tested further *in vitro* for functionality in Phe activation (Fig. 3, Table 2). The wild‐type *Hsmt*PheRS and the Pro49Ala mutant both stimulate Phe‐dependent ATP hydrolysis with nearly identical specific activities. While an approximately twofold reduction in the ATP consumption rate was seen in the His99Asp mutant, this dropped to a 20‐fold reduction in the Arg117Gly, Gly273Ser, and His123Pro mutants. Notably, the effects on the *Hsmt*PheRS activity in Phe activation in the His99Asp, His123Pro and Gly273Ser mutants did not correlate with their efficiency of tRNA^Phe^ aminoacylation (Tables 1 and 2). All three of these mutations were impaired the aa activation (Fig. 3) to a lesser extent than in the transfer of the amino acid group to the tRNA. The Arg117Gly and His123Pro mutations have comparable effects on the *Hsmt*PheRS activity at both steps of the aminoacylation reaction.

**Table 2:** Specific Activities of HsmtPheRS Mutants in the Phe Activation Reaction

*Hsmt*PheRS	Initial velocity (µ*M*/min)	Initial velocity (relative)
WT	0.54 ± 0.05	1
P49A	0.66 ± 0.05	1.2
H99D	0.21 ± 0.03	0.39
R117G	0.02 ± 0.002	0.042
H123P	0.02 ± 0.002	0.043
G273S	0.04 ± 0.003	0.06

The initial velocities of the activation reaction were determined by ATP consumption assay at identical concentrations of WT and mutant enzymes (0.8 µ*M*).

### Crystal structures

To determine whether mutations triggered conformational changes in *H*s*mt*PheRS, we cloned all the mutant sequences, expressed and purified the recombinant proteins without His‐tags in preparation for crystallization studies using conditions analogous to those used for WT *H*s*mt*PheRS.^22^ Only four mutants out of nine generated crystals, which diffracted to 1.87 Å, 1.89 Å, 2.05 Å, and 1.46 Å resolution for Pro49Ala (PDB ID 5MGH), Thr210Met (PDB ID 5MGU), Asp289Tyr (PDB ID 5MGV), and Arg383Cis (PDB ID 5MGW), respectively (Table 3). The crystal structures of these four mutants resembled each other in the overall molecular structure and all of them are closely related to the three‐dimensional structure of WT *H*s*mt*PheRS. Upon superimposition of the mutant crystal structures onto that of WT *H*s*mt*PheRS, they display the following values of r.m.s.d.: 0.256 Å over 347 Cα atoms for Pro49Ala; 0.262 Å over 368 Cα atoms for Thr210Met; 0.194 Å over 364 Cα atoms for Asp289Tyr; and 0.442 Å over 345 Cα atoms for Arg383Cys. The failure to crystallize Arg117Gly, Gly273Ser, His99Asp, and His123Pro mutants may be related to the significant conformational changes that their 3D structures undergo. There is a straightforward interpretation of the results as they immediately relate to the successive snapshots of MD simulations carried out for each of the mutants. Rearrangements in the topology of the molecule prevent formation of intermolecular contacts, required for crystallization and the availability of the long range order in the crystals. For mutant Arg387Glu we could see only microcrystals, and failed to get crystals suitable for X‐ray data collection.

**Table 3:** Data Collection and Refinement of HsmtPheRS Mutants

	Pro49Ala	Thr210Met	Asp289Tyr	Arg383Cys
Resolution range (Å)	47.9–1.87 (1.94–1.87)	46.66–1.89 (1.96–1.89)	50.05–2.05 (2.08–2.05)	45.9–1.46 (1.512–1.46)
Space group	P 2_1_ 2_1_ 2_1_	P 2_1_ 2_1_ 2_1_	P 2_1_2_1_2_1_	P 2_1_ 2_1_ 2_1_
Cell dimensions *a*, *b*, *c* (Å)	55.04, 89.35, 97.23	54.54, 90.13, 97.45	54.77, 90.37, 95.17	53.35, 90.12, 99.08
*α*, *β*, *γ* (°)	90, 90, 90	90, 90, 90	90,90,90	90, 90, 90
Unique reflections	40,252 (3917)	38,365 (3749)	28,291 (1373)	83,388 (8212)
Completeness (%)	100	98	93.2	100
Mean *I*/sigma (I)	25.2	22.14	25.22	33.2
Wilson *B*‐factor (Å^2^)	35.84	37.27	25.15	21.09
*R*‐merge	7.2	6.8	7.0	5.1
Reflections used in refinement	40,241 (3913)	38,356 (3746)	26,918 (1373)	83,373 (8208)
Reflections used for *R*‐free	2003 (204)	1999 (195)	679 (40)	2000 (197)
*R*‐work	0.1976 (0.3070)	0.2024 (0.3579)	0.2089 (0.2574)	0.1851 (0.3112)
*R*‐free	0.2299 (0.3436)	0.2415 (0.4322)	0.2460 (0.3061)	0.2050 (0.3326)
No. nonhydrogen atoms	3622	3611	3607	3887
Water molecules	259	235	249	522
Protein residues	405	407	405	406
RMS (bonds) (Å)	0.007	0.007	0.007	0.006
RMS (angles) (°)	0.91	1.00	1.2	0.82
Ramachandran favored (%)	96	95	95	97
Ramachandran allowed (%)	3.5	4.4	3.8	3.0
Ramachandran outliers (%)	0.99	0.49	1.1	0
Average *B*‐factor	42.67	42.51	46.5	28.25
Macromolecules	42.36	42.21	39.8	26.93
Solvent	46.71	46.77	48.8	36.75

### Mutant Asp289Tyr

**Figure 4:**
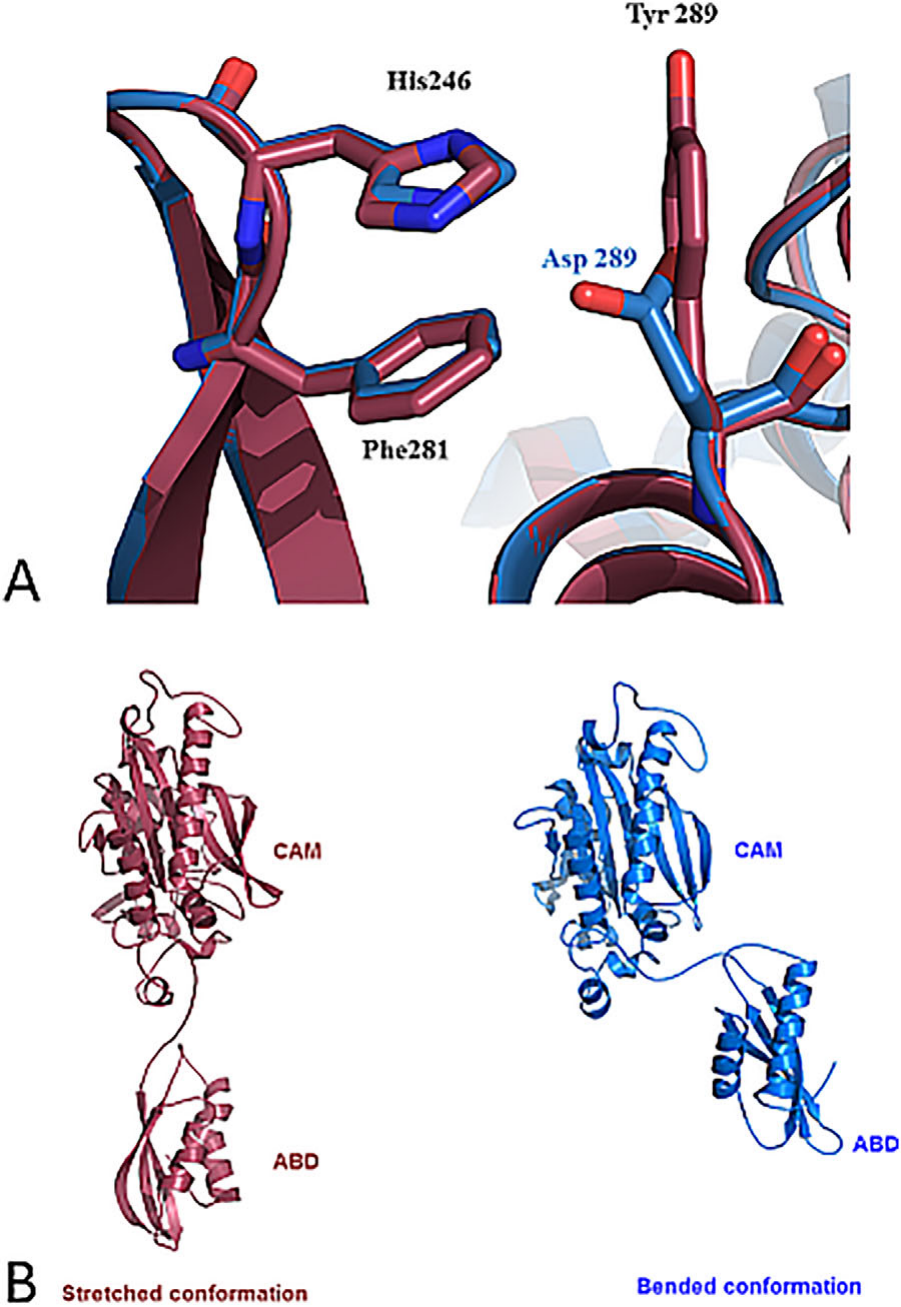
(A) Superimposition of the crystal structure of WT *Hsmt*PheRS (blue) on to the mutant Asp289Tyr structure (burgundy) at the intersubunit interface. The perfect alignment of 3D‐structures is clearly seen. Electrostatic interaction between His246 and Asp289 is substituted in this area for edge‐to‐face interactions between Phe281 and His246 from one side and Tyr289 from the other side. (B) Snapshots of global molecular dynamic simulations performed for the WT and Asp289Tyr mutant forms of *Hsmt*PheRS (blue and burgundy, respectively) stopped at 22 ns on the pathway trajectory. Upon repositioning of the ABD from “closed” to an “open” state, WT molecule and Asp289Tyr mutant are passing through a number of similar intermediate states (mutation on the outer surface of the enzyme). Due to slowing down of ABD relocation in mutant, the WT and Asp289Tyr will go through similar conformations, but at different moments of time.

Patient 5M (Supporting Information Table S1) is homozygous for Asp289Tyr in the *FARS2* gene encoding *Hsmt*PheRS. Patient 5M harbored a partial genomic deletion and a highly conserved Asp325Tyr missense variant (the difference in aa numbering is −36) that caused early‐onset epilepsy and an isolated complex IV deficiency in muscle.^6^ Within *Hsmt*PheRS, Asp289 is located at the interface of CAM and ABD. The mutated Asp289Tyr located at a distance from the synthetic active site and the contact area with cognate tRNA. The mutant demonstrates, however, a twofold reduction in the catalytic efficiency, due to a decreased *k*_cat_ value (Table 1). Whilst no initial evidence suggests that this mutation is responsible for the observed reduction of the catalytic constant, we crystallized the mutated *Hsmt*PheRS in the complex with Phe and ATP, as previously described^13^, ^22^ to identify its effect on 3D structure. The crystals diffracted to 2.0 Å resolution and belong to the space group P2_1_2_1_2_1_, with unit cell parameters of *a* = 54.9 Å, *b* = 90.0 Å, *c* = 96.0 Å (see Materials and Methods). The final atomic model includes 405 aa residues and 250 water molecules (Table 3). *Hsmt*PheRS superimposed onto the WT *Hsmt*PheRS with r.m.s.d. 0.24 Å over 400 C_α_ atoms. The X‐ray data revealed a prominent structural distinction between the mutant *Hsmt*PheRS and the WT enzyme in the appearance of the edge‐to‐face interactions between pair of Phe245 and His246 from the one side and Tyr289 from the other [Fig. 4(A)]. Thus, a new strong interaction between modules CAM and ABD is added to the mutated variant.

In order to uncover the reason of the activity reduction, we performed molecular dynamic (MD) simulations both for the WT *Hsmt*PheRS and Asp289Tyr mutant. The initial set of coordinates for the two molecules corresponded to the open state of *Hsmt*PheRS, as experimentally observed in the complex with cognate tRNA^Phe^.^13^ Simulations for WT and mutant were performed for 50 ns (see Materials and Methods) and revealed that the WT enzyme generates three major clusters of inter‐domain contacts. These are salt bridges between ABD and CAM that are generated upon transitions from an open conformation to a closed one. The first contact is formed between Arg302 and Glu358 that belong to CAM and ABD, respectively. This bridge stabilizes open conformations upon formation of the complex with tRNA, and remains intact during almost half of the total MD simulations period (∼20 ns). Subsequently, the Arg302 switches from Glu358 to Asp355, forming the next salt bridge in the cascade of contacts. After ∼40 ns the ABD moves along its trajectory towards the closed conformation by breaking previous contacts with Arg302 and generating new ones. The Asp289Tyr mutant displayed substantial differences in dynamic behavior and mechanical trajectory [Fig. 4(B)]. The conclusion from the MD simulation is that the transition from the open “active” conformation to the closed “inactive” one happens almost approximately eight times faster (∼6 ns) in the Asp289Tyr mutant. This led us to a remarkable finding: statistically the mutated enzyme spends longer time in the closed inactive state, thereby reducing the potential for aminoacylation activity of *Hsmt*PheRS, consistent with the activity measurements. Thus, an *in silico* MD approach appears to be very useful in revealing functional roles of the mutations whose detrimental effects on the catalytic efficiency are otherwise difficult to describe in terms of structure and function.

### Mutant Arg117Gly

This was one of two heterozygous variants, c.457A > G (p.Arg153Gly; in 3DN is Arg117Gly) and c.925G > A (p.Gly309Ser; in 3DN is Gly273Ser) identified in the human *FARS2* gene (NM_006567.3) of Patient 1F (Supporting Information Table S1), both were predicted to be deleterious by SIFT and PolyPhen2.^3^ This baby girl was 5 months old at the time of diagnosis, presenting with poor growth, persistent lactic acidosis with ketosis.

**Figure 5:**
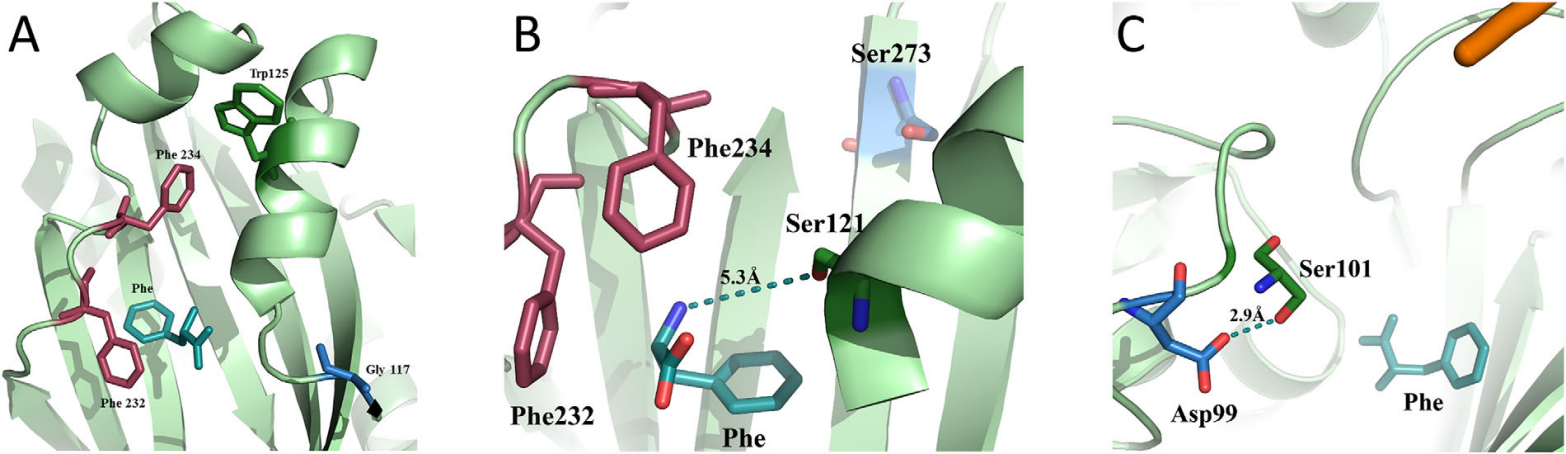
Structural, dynamic and functional characterization of the Arg117Gly, Gly273Ser, and His99Asp mutants of *Hsmt*PheRS. (A) The diagram depicts the rearrangement of the aa residues within the active site. After 24 ns of MD simulation the Phe234 had migrated from its standard position toward Trp125, thereby disrupting the aromatic triad forming a “network” of edge‐to‐face interactions. The Phe substrate is colored light green; (B) the active site Phe bound is colored forest green. The distance between the amino group of the ligand and Ser121 after 18 ns of MD simulation is depicted by a dashed line. Phe232 and Phe234 that provide specific recognition of Phe substrate are colored burgundy; (C) depicted is the appearance of HB between Ser101 and Asp99 on the MD simulation trajectory. As can be seen on the snapshots of MD simulations, the position of the Phe substrate is slightly altered.

The residue Arg117 is located in the catalytic core of *Hsmt*PheRS where its guanidinium group makes an internal salt bridge with Asp140 (in the strand) and HB with His123 (within the same helix). It constitutes a principal structure‐forming contact of the CAM topology by holding together and correctly spacing the flanking helices and strands. Mutation of Arg to Gly will disturb the salt bridge between Asp140 and Arg117, thereby triggering significant changes in the CAM conformation. Moreover, appearance of glycine in the Leu116‐Thr120 loop involved in the Phe‐AMP intermediate formation, may lead to a reduction in the enzyme activity due to flexibility of the loop. Even after ∼6 ns of MD simulation, the Phe234, which together with the Phe substrate and Phe232 forms an edge‐to‐phase recognition triplet, escapes its position in the mutant and moves towards Trp125 [Fig. 5(A)]. The trajectory of Trp125 motion is very long, generating at the end a new edge‐to‐phase interaction with Phe234. This rearrangement halves the binding energy of Phe compared to the WT *Hsmt*PheRS (missing interaction between substrate Phe and Phe234) and leads to a substantial reduction in Phe activation and aminoacylation activity (Tables 1 and 2). The steady‐state aminoacylation kinetics of the Arg117Gly mutant showed a ∼50‐fold reduction in the *k*_cat_/*K*_m_ value as a result of an increased *K*_m_ value for Phe and a decreased *k*_cat_ in comparison with WT enzyme (see Table 1). A 24‐fold reduction in the rate of ATP consumption caused by the mutation was also observed (Table 2).

### Mutant Gly273Ser

Patient 1F (Supporting Information Table S1) is heterozygous for Arg153Gly (3DN is Arg117Gly) and p.Gly309Ser (Gly273Ser in 3DN; p.Arg117Gly is described above).^3^ Gly273 residue belongs to the β‐strand Asp270‐Gly279, one of the shape‐generating elements of CAM. The glycine‐lined strand contacts the reaction intermediate Phe‐AMP^23^ and the emergence of serine instead of glycine will result in steric clashes between the side chains of serine and Met258 (∼2 Å). This in turn will lead to reorganization of the hydrophobic cluster in which Met258 holds the central position. MD simulation of the mutant reveals that Ser273 causes formation of a new very stable HB with Gln124 (2.8–3.3 Å), which holds up ∼95% of the whole cycle of MD trajectory. This contrasts with the WT *Hsmt*PheRS, where this contact is ∼9 to 10 Å.

Anchoring the amino group of the Phe‐substrate is achieved by its interactions with Ser121, and His119 via direct or water‐mediated contacts.^13^, ^14^, ^15^ The analysis of MD snapshots in the Gly273Ser mutant demonstrates loss of contacts between residues Ser121 and Glu159, and the amino group of the Phe substrate. The latter moves by 5 to 6 Å away from its original position thereby, rendering impossible the activation process and subsequent aminoacylation (Fig. 3). Moreover, within ∼7 ns of MD trajectory the canonical “edge‐to‐face” aromatic interactions were lost and there was evidence that the binding of the Phe substrate was also lost [Fig. 5(B)]. The kinetic experiments showed that mutant is not charging tRNA^Phe^ at the same concentrations as WT *Hsmt*PheRS (Table 1). Activation and aminoacylation activity of this Gly273Ser mutant (Tables 1 and 2) could only be detected at very high enzyme concentrations.

### Mutant His123Pro

Patient 3F is a 17‐year‐old girl (Supporting Information Table S1) harboring a p.His159Pro (3DN is His123Pro) change from the wild‐type human *FARS2* gene (NM_006567.3). She presented with developmental delay, encephalopathy, elevated CSF lactate, and abnormal respiratory enzymes, consistent with the clinical features reported for *FARS2* defects. Two compound heterozygous variants, His123Pro (from the mother) and Arg383Cys (from the father) were identified both of which were predicted to be deleterious by SIFT and PolyPhen2.^3^

The complex of *Hsmt*PheRS with tRNA^Phe13^ suggests that the His to Pro substitution leads to considerable reorganization of the structure. First, the HB between Arg117 and His123 will be disrupted; second, the presence of the His123Pro mutation will break the α‐helical structure (His119‐Ala130). Since the α‐helical residues make direct contact with the Phe‐AMP, it is unsurprising that this mutation leads to a dramatic reduction in the enzymatic activity (Table 1). Our data confirm that the His123Pro mutant has lost the ability to catalyze Phe‐tRNA^Phe^ production at concentrations that give detectable activity by WT *Hsmt*PheRS. We estimated its catalytic efficiency to be 3100‐fold lower than that of the wild type enzyme (Table 1). All crystallization trials for the mutant failed, as a result of the significant changes in the folding capacity of CAM.

### Mutant Pro49Ala

Patient 2F (Supporting Information Table S1), a 16‐year‐old girl, is heterozygous for two variants in *FARS2* (NM_006567.3), c.253C > G (Pro49Ala), and c.403C > G (His99Asp), both predicted to be deleterious by SIFT and PolyPhen2.^3^ A more detailed clinical investigation of this mutation has recently been published.^24^

Pro49 belongs to a fully hydrophobic nucleus located between the ABD and CAM that is next to a long flexible stretch of aa, Asn313‐Pro325, connecting the two structural modules. Substitution for alanine does not disrupt the hydrophobicity of this area and the mutation would not initially be expected to exert a direct impact on Phe activation or tRNA aminoacylation since it is located at a distance from the active site. MD simulation demonstrates that substitution of Pro49 for Ala changes the movement of ABD along the trajectory towards the CAM. During the first 20 ns of dynamic fluctuations the system moves towards the “closed” form and remained in this inactive conformation for an extended time, thus causing impaired aminoacylation activity through a similar mechanism to that observed for the Asp289Tyr mutant. The WT *Hsmt*PheRS and the Pro49Ala mutant stimulate Phe‐dependent ATP hydrolysis with nearly identical specific activities (Table 2). The kinetic study of tRNA^Phe^ charging revealed that due to a reduced *k*_cat_ there was only a 1.3‐fold decrease in the *k*_cat_/*K*_m_ value (see Table 1) suggesting that the P49A mutant charges tRNA^Phe^ in a way similar to the WT, which correlates well with data in Table 1.

### Mutant His99Asp

Patient 2F is heterozygous (NM_006567.3) for c.403C > G (3DN is His99Asp; see previous paragraph).^3^, ^24^ The previously published structures of *Hsmt*PheRS with tRNA^Phe^ or Phe‐AMP^10^, ^13^ indicate that His99 residue is located in the immediate vicinity of the 3’‐end of the tRNA. The 3’‐end of tRNA^Phe^ is virtually clamped between the first intermediate, Phe‐AMP, and the Leu94‐Gly105 fragment of the CAM. The His99 side chain is exposed towards the 3’‐end of the tRNA and since, the tRNA molecule is negatively charged,^20^, ^25^ the appearance of Asp99 instead of His99 may result in local destabilization of the whole complex: *Hmst*PheRS•tRNA^Phe^•Phe‐AMP. One can hypothesize that substitution of the positively charged His99 by the negatively charged aspartic acid will result in repulsion of the acceptor stem from its correct position, where the tRNA is charged with its cognate aa. Analysis of the MD snapshots of the mutant demonstrated that Asp99 forms a stable HB (∼3 Å) with Ser101 [Fig. 5(C)] that exists ∼65% of the entire travel time on the MD trajectory, thereby stabilizing this new conformation. The Side chain of a negatively charged Asp99 in mutant generates additional repulsion from tRNA^Phe^ (also negatively charged) on its pathway to the binding site. The MD snapshots demonstrate that HB between the amino group of Phe‐substrate and Ser119, essential for Phe‐AMP formation, occurs less frequently as compared to WT *Hsmt*PheRS, thus explaining the significant reduction in the aminoacylation activity (Table 1).

A 40‐fold reduction in the *k*_cat_/*K*_m_ for His99Asp variant results from a combined increased *K*_m_ value for Phe and a decreased *k*_cat_ value and led to a 2.6‐fold reduction in the rate of ATP consumption (Table 2). Notably, the impact of the His99Asp mutation on Phe activation (Fig. 3, Table 1) does not correlate with the impact on the catalytic efficiency of tRNA^Phe^. Substitution of His for Asp impairs the aa activation step to a lesser extent than the transfer step of Phe from Phe‐AMP to tRNA^Phe^ (Tables 1 and 2). The activity of His99Asp mutant presented by Walker et al.^24^ was not detected in aminoacylation reaction due to low enzyme concentration used in experiments. We were able to detect this activity only at very high enzyme concentration. Data for activation reactions correlate well with results presented in Ref. 22.

### Mutant Arg387Gln

Patient 4M (Supporting Information Table S1) is a 4‐year‐old boy who presented with non‐specific developmental delay and hypotonia and was found to have two heterozygous changes (c.737C > T (p.Thr246Met; 3DN is Thr210Met) and c.1268G > A (p. Arg423Gln; 3DN is Arg387Gln)) to the human *FARS2* (NM_006567.3) gene. SIFT and PolyPhen2 predicted Arg387Gln to be deleterious, but made a discordant prediction regarding the pathogenicity for Thr210Met.^3^

**Figure 6:**
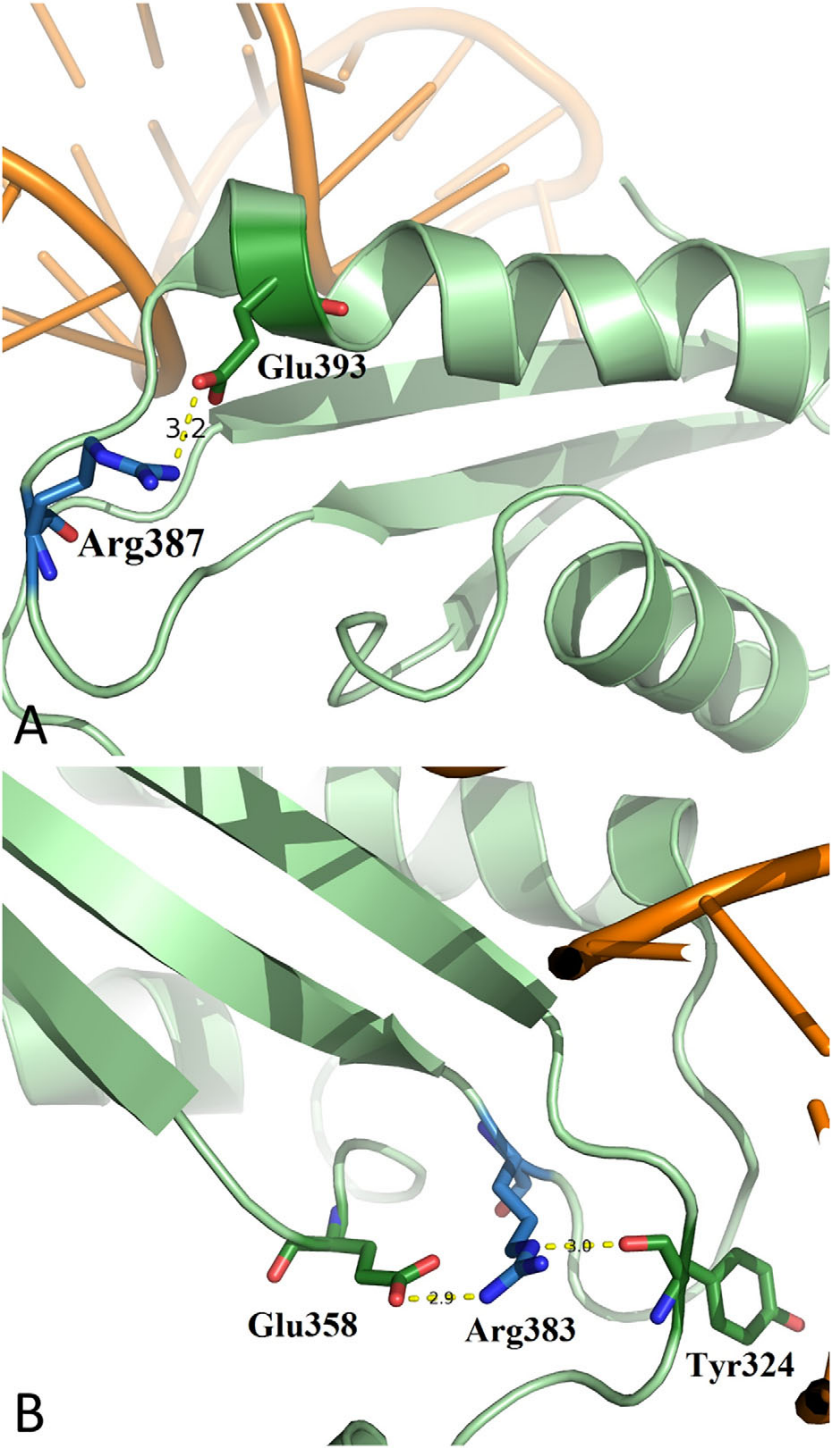
Structural and functional characterization of the Arg387Gln and Arg383Cys mutants of *Hsmt*PheRS. (A) Diagram depicting HB formation between residues Arg387 and Glu393, stabilizing the conformation of the ABD, and connecting basic structural elements (two strands) within the ABD of WT *Hsmt*PheRS. (B) Diagram showing HB formation between aa residues of two adjacent strands of the ABD in the WT *Hsmt*PheRS. Arg383 is a shape‐generating residue of ABD, makes HB with Glu358 and the carbonyl group of Tyr324 (acceptor).

Arg387 is located in the ABD, is exposed to solution and the crystal structure of *Hsmt*PheRS in complex with tRNA^Phe^ indicates that Arg387 has no direct or indirect contacts with bound tRNA^Phe^ [Fig. 6(A)].^13^ The substitution of Arg387 for glutamine breaks a salt bridge between Arg387 and Glu393. The substituted Gln387, however, also has a propensity to form HB since the amide group can accept and donate a hydrogen atom. Thus, Gln387 and Glu393 may continue to maintain the HB and support conformation of the Tyr378‐Ser385 loop and helix. Comparing the dynamic behavior of WT *Hsmt*PheRS with that of the Arg387Gln mutant, we could conclude that catalytic efficiencies for these two variants appear to be similar and the kinetic experiments showed only a 1.2‐fold decrease in the *k*_cat_/*K*_m_ value due to a reduced *k*_cat_ (Table 1).

### Mutant Arg383Cys

Two siblings who are compound heterozygotes for the variants Arg383Cys and an intragenic deletion have a comparatively moderate presentation among the spectrum of *FARS2* related phenotypes.^4^ Clinical features in these siblings include truncal hypotonia, global developmental delays, mild facial dysmorphia, and variable lactic academia. Another patient with the Arg383Cys mutation (compound heterozygous His123Pro from the mother; Arg383Cys from the father) is a 17‐year‐old girl (Patient 3F) who displayed developmental delay, encephalopathy, elevated CSF lactate and abnormal respiratory enzymes, consistent with reported clinical features for *FARS2* defects.

Arg383 is a shape‐generating residue of ABD, making HB with Glu358 [Fig. 6(B)] and the carbonyl group of Tyr324 (acceptor). A given net of HB contributes significantly to stabilizing the ABD fold by cross‐linking three β‐strands that form the inner platform of ABD. Substitution of Arg383 for cysteine may partially disrupt a salt bridge between Arg383 and Glu358, however, this mutation will not have a harmful effect on the anticodon recognition or aminoacylation of tRNA^Phe^, and since protonated Glu358 can form HB with Cys. The Arg383Cys mutation leads to an approximately twofold reduction in the catalytic efficiency (Table 1) as the *k*_cat_/*K*_m_ is reduced due to a 1.8‐fold increase of *K*_m_ and a 1.3‐fold decrease of *k*_cat_.

### Mutant Thr210Met

Patient 4M (Supporting Information Table S1) is a 4‐year‐old boy with two heterozygous variants to the *FARS2* gene c.737C > T (Thr210Met) and c.1268G > A (Arg387Gln) (p.T246M in 3DN is Thr210Met) who presented with nonspecific developmental delay and hypotonia. SIFT and PolyPhen2 predicted Arg423Gln to be deleterious but made a discordant prediction regarding the pathogenicity for Thr210Met.^3^

Residue Thr210 is located within the CAM but has no direct contacts with either tRNA^Phe^ or the Phe‐AMP intermediate. The Thr210Met substitution would not a priori be predicted to change the mutant *Hsmt*PheRS architecture or the ability for Phe‐AMP complex formation. The dominant mode after 30 ns of MD simulation showed that the relative position of CAM and ABD is similar to that observed for the crystal structure of WT *Hsmt*PheRS complexed with tRNA^Phe^ [Fig. 2(A)]. Replacement of Thr210 by Met enhances hydrophobic interactions with Phe61, Tyr65, Phe74, and Trp274 and led to a ∼1.4‐fold increase in the catalytic efficiency of tRNA^Phe^ charging, due to a 3.3‐fold decrease in *K*_m_ value for tRNA^Phe^ (see Table 1).

## Discussion

Here, we present the in‐depth analysis of changes in the 3D structure and kinetic characteristics of *Hsmt*PheRS, induced by pathogenic mutations in the *FARS2*. Analysis of high resolution crystal structures of WT *Hmst*PheRS and those complexed with different functional ligands enables to follow the reaction pathway by which *Hsmt*PheRS catalyzes attachment of Phe to tRNA^Phe^. The positions of the residues that are critically important for the arrangement and binding of Phe, ATP, tRNA substrates, and for enzyme activity were established.^10^, ^13^, ^26^, ^27^ Data on the WT *Hsmt*PheRS and mutants derived from kinetic experiments, X‐ray crystallography and MD simulations are indicative of direct relationship between conformational changes in 3D structure and *k*_cat_/*K*_m_ values of tRNA^Phe^ aminoacylation and/or specific activity in the ATP consumption.

The most deleterious effect on enzymatic activity were caused by the His99Asp and Arg117Gly mutations, which demonstrated a 40‐ to 50‐fold decrease in catalytic efficiency of aminoacylation. Furthermore the kinetic assays revealed that the Arg117Gly mutation also led to a 24‐fold reduction in the rate of ATP consumption, whereas the effect of the His99Asp substitution was more modest with only a 2.6‐fold reduction (see Tables 1 and 2). This information will be used more extensively to determine any correlation between the molecular pathogenesis and the clinical manifestation. The expanding resources resulting from exome and whole genome sequencing together with increased characterization of proteins has confirmed the importance of pathogenic mutations in mitochondrial aaRSs as the cause of many neurological disorders. The crystal structures of mutant versions of *Hsmt*PheRS, together with the kinetic data and MD simulations that are presented here exemplify how mutations can affect the overall structure in ways that would not be predicted from 3D structure only.

It was inferred that the severity of the disease and the specific tissue phenotypes that was caused by the different site of mutations of the same gene might depend on residual enzymatic activity or structural instability (see Tables 1 and 2). It is worth mentioning that all of the reported enzyme activities were detected via *in vitro* assays that might have some slight difference *in vivo*. Future comprehensive documentation of the clinical course of mitochondrial disease arising from specific mutations in the aaRSs together with detailed structural analyses will facilitate a better understanding of the molecular mechanism underlying the pathogenesis, which may in turn have predictive therapeutic potential.

## Materials and Methods

### tRNA aminoacylation

Aminoacylation reactions were performed at 37°C in reaction mixtures containing 50 m*M* Tris‐HCl (pH 8.5), 30 m*M* MgCl_2_, 5 m*M* 2‐mercaptoethanol, 5 m*M* ATP, 0.5–3 μ*M* [^14^C/^3^H]‐labeled l‐Phe, and 0.5 to 3 μ*M Escherichia coli* tRNA^Phe^ transcript. The enzyme concentration varied from 0.15 μ*M* for the wild‐type *Hsmt*PheRS to 1.5 μ*M* for mutants. At the appropriate time points aliquots (4 µL) were spotted onto Whatman filter paper impregnated with 5% TCA. The filters were then extensively washed with ice‐cold 5% TCA, and TCA‐insoluble radioactivity was measured by liquid scintillation counting. The kinetic parameters were calculated by a nonlinear regression fit of the data to a Michaelis‐Menten equation. The reported *k*_cat_ and *K*_m_ values represent the average of at least two determinations with experimental errors within 15 to 20% of the indicated values.

### ATP Hydrolysis Assay

Formation of aminoacyl‐adenylate was directly measured by means of thin‐layer chromatography.^28^ The reaction mixture contained 50 m*M* Tris‐HCl (pH 8.0), 30 m*M* MgCl_2_, 5 m*M* 2‐mercaptoethanol, 1 m*M* l‐phenylalanine, 30 μ*M* [α‐^32^P]ATP, and 10 U/mL of inorganic pyrophosphatase. The reaction, performed at 37°C, was initiated by adding 150 to 300 n*M* PheRS (wild‐type or mutant). ATP, Phe‐AMP, and AMP (formed during hydrolysis of Phe‐AMP) were separated by TLC (on PEI cellulose plates, Merck) developed in a mixture of acetic acid, 1M ammonium acetate, and water (5:10:85, v/v). The radioactivity of spots was quantified by PhosphorImaging.

### MD Simulation

The MD simulations of WT enzyme and the mutants variants were conducted using GROMACS^29^ version 4.5.5. The multistep simulation protocol includes the following stages. The Steepest Descent technique (1000 steps) has been applied in a vacuum of the mutated residues only, while keeping other atoms restrained at their initial positions.

Water molecules were modeled as single point charges (SPCs). Minimized structures were placed at the center of a SPC water box. The total electric charge of each protein‐water system was neutralized by sodium counter ions added to the system. The energy of the solvated structures was then minimized by using the steepest descent method (5000 steps) followed by conjugate gradient minimization (10,000 steps). To achieve better relaxation for initial configurations, a 100 ps MD simulation was performed at constant pressure and temperature *T* (300 K). The positions of protein nonhydrogen atoms were restrained by a force constant of 1000 kJ mol^−1^ Å^−1^. Bond lengths were restrained using the LINCS algorithm^30^ applied with the 2 fs integration step, and the neighbor list for calculation of nonbonded interactions was updated every five time steps. Periodic boundary conditions were used, and electrostatic interactions were calculated by using the PME method^31^ with a short‐range cutoff of 1.0 nm. For the Lennard–Jones interactions, a cutoff value of 1.0 nm was used.

Trajectories (30–100 ns for each mutant) were sampled at a constant pressure (1 bar) and temperature (300 K) using the Berendsen thermal bath.^29^ A convergence and reproducibility of mutant's internal motion has been tested on trajectories applying principal component analysis.^32^

## Supporting information

Supporting Information
