# Supplementary material for: Kinetic and structural changes in H smt PheRS, induced by pathogenic mutations in human FARS 2
Source: Protein Sci. 2017 May 3;26(8):1505–16. doi: 10.1002/pro.3176 (PMC5521548; doi:10.1002/pro.3176)
Supplement: Supplementary file 1 — Supporting Information [file PRO-26-1505-s001.docx]

**Table S1**

**Molecular and clinical findings of FARS2**

| Patient-gender | Age | Allele 1 | Kcat/Km  relative | Allele 2 | Kcat/Km  relative | Additive | Clinical phenotype | Pathogenic correlation |
| --- | --- | --- | --- | --- | --- | --- | --- | --- |
| WT |  |  | 1 |  | 1 |  |  |  |
| 1 F | 0.4 | R117G | 0.021 | G273S | 0.00025 | 5x10^-6^ | Poor growth, persistent lactic acidosis with ketosis | yes |
| 2 F | 15.5 | P49A | 0.78 | H99D | 0.027 | 0.0034 | Not provided |  |
| 3 F | 16.8 | H123P | 0.00032 | R383C | 0.43 | 0.00014 | Development delay, encephalopathy, migraines, perinatal insult, pyramidal signs, spasticity, dystonia, high CSF lactate, elevated pyruvate, elevated alanine, gonadal failure, abnormal respiratory enzymes | yes |
| 4 M | 4 | T210M | 1.4 | R387Q | 0.86 | 1.2 | Nonspecific phenotype developmental delay, hypotonia, speech delay | yes |
| 5 M | 0.5 | D289Y | 0.52 |  |  |  | Early-onset epilepsy and isolated complex IV deficiency in muscle. A mild intermittent seizure disorder,  spastic paraplegia. | yes |
